# The Evolution of Triamcinolone Acetonide Therapeutic Use in Retinal Diseases: From Off-Label Intravitreal Injection to Advanced Nano-Drug Delivery Systems

**DOI:** 10.3390/biomedicines11071901

**Published:** 2023-07-05

**Authors:** Luis Abraham Aceves-Franco, Oscar Eduardo Sanchez-Aguilar, Allen Rafael Barragan-Arias, Marco Antonio Ponce-Gallegos, Jose Navarro-Partida, Arturo Santos

**Affiliations:** 1 Tecnologico de Monterrey, Escuela de Medicina y Ciencias de la Salud, Monterrey 64849, Nuevo Leon, Mexico; a00832512@tec.mx (L.A.A.-F.); a01206795@tec.mx (O.E.S.-A.); a01637937@tec.mx (A.R.B.-A.); marcoapg31@gmail.com (M.A.P.-G.); josenavarro@tec.mx (J.N.-P.); 2 Centro de Retina Medica y Quirurgica, S.C., Centro Medico Puerta de Hierro, Zapopan 45116, Jalisco, Mexico

**Keywords:** triamcinolone acetonide, retinal diseases, posterior segment disease, nano delivery systems, liposomes, intravitreal injections

## Abstract

Ophthalmic drug delivery to the posterior segment of the eye has been challenging due to the complex ocular anatomy. Intravitreal injection of drugs was introduced to deliver therapeutic doses in the posterior segment. Different posterior segment diseases including age-related macular degeneration, diabetic macular edema, retinal vein occlusions, uveitis, and cystoid macular edema, among others, have been historically treated with intravitreal corticosteroids injections, and more recently with intravitreal corticosteroids drug implants. Triamcinolone acetonide (TA) is the most frequently used intraocular synthetic corticosteroid. Using nanoparticle-based TA delivery systems has been proposed as an alternative to intravitreal injections in the treatment of posterior segment diseases. From these novel delivery systems, topical liposomes have been the most promising strategy. This review is oriented to exhibit triamcinolone acetonide drug evolution and its results in treating posterior segment diseases using diverse delivery platforms.

## 1. Introduction

Topical ophthalmic cortisone was used in the 1950s to treat inflammatory conditions of the anterior segment of the eye. However, topical ophthalmic cortisone did not show therapeutic effects on retinal and posterior segment diseases [1]. In 1974, intraocular injections of dexamethasone started being administered in animal models to overcome these challenges [2]. In 1980, Tano et al. reported using Triamcinolone acetonide (TA) to treat intraocular proliferative fibroblastic processes generated after surgery in rabbits [3], suggesting the possible advantages of this medication through different routes of administration. TA is a synthetic steroid and is considered the prototypic corticosteroid for the treatment of posterior segment diseases. It has an increased anti-inflammatory potency, having a five-fold higher glucocorticoid activity than hydrocortisone with a minimal percentage of mineralocorticoid agonist activity [4,5,6]. TA is commercially available as an ester presenting minimal water solubility but complete solubility in alcohol and is characterized by its ability to remain for longer periods in the vitreous cavity [6,7,8,9,10]. TA has a mean half-life of 15 days; however, trials have shown that vitrectomized and aphakic eyes dramatically decrease the average duration of remaining TA in the eye [11,12,13,14,15,16].

Intravitreal administration of TA (IVT TA) has shown a great impact in the treatment of different posterior segment diseases, including age-related macular degeneration (AMD), cystoid macular edema (CME), diabetic macular edema (DME), and edema related to central retinal vein occlusion [17,18]. This drug is not exempt from adverse effects, such as intraocular hypertension and cataract formation. In addition, several complications have been related to this route of administration, such as intraocular infection, retinal detachment, and lens injury [19]. Preservative-containing TA formulations have been shown to produce retinal toxicity after their administration, leading to the development of preservative-free TA formulations [20]. Antiangiogenic drugs have displaced the use of steroids for vitreoretinal diseases and have become the gold standard treatment for these pathologies [21]. Nevertheless, treatment with both TA and antiangiogenic drugs could result in more beneficial effects rather than single monotherapy [22]. Owing to the benefits of TA, several corticosteroid implants have been developed and approved for intraocular use. Currently, thorough research in nano-formulations containing TA has increased the possibility of better treatment approaches, especially with the improvement of drug delivery systems for topical formulations [23]. In this review, we explore the administration routes, biological effects, available formulations, adverse effects, therapeutic applications in posterior segment diseases, and innovative administration methods of TA to understand its therapeutic impact on vitreoretinal diseases.

## 2. Routes for Ocular Administration of TA

**Figure 1:**
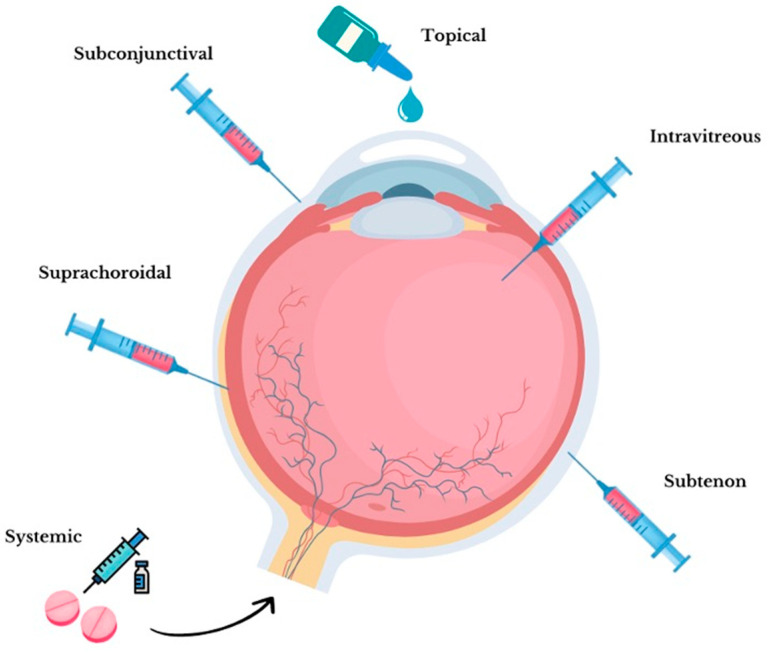
Triamcinolone acetonide routes of administration.

The routes of TA administration have become one of the main challenges in ocular pharmacology. The ocular globe is separated anatomically into two separate segments, the anterior and posterior. The anterior segment includes the cornea, iris, ciliary bodies, and lens and includes all the spaces filled with the aqueous humor. The posterior segment extends behind the lens and includes the retina, choroid, and optic nerve, and the vitreous cavity filled with the vitreous gel [24]. The difference between the anterior and posterior segments of the eye has forced the development of topical, intravitreal, and periocular (suprachoroidal, subtenon, and subconjunctival) injections, and systemic (parenteral, oral) TA formulations to achieve greater efficacy and minimize adverse effects in the treatment of their respective segmental diseases. A summary of the different routes of TA ocular drug delivery is depicted in Figure 1.

Topical TA formulations, such as eye drops, represent one of the preferred routes of administration due to several benefits such as fewer adverse effects, lower costs, and greater patient compliance. However, its usefulness is limited for treating diseases of the posterior segment of the eye due to the complex eye anatomy and its dynamic barriers such as tear drainage, conjunctival clearance, and choroidal and lymphatic circulation. It is known that less than 5% of the topically administered drug reaches the posterior segment of the eye [25,26]. Intravitreal injections have been the preferred method for TA administration to the posterior segment of the eye due to the increased concentration, bioavailability, and efficacy they offer. However, this form of administration is considered an invasive procedure and is associated with severe complications such as endophthalmitis, lens injury, and retinal detachment. These therapies are also expensive owing to the requirement for monthly dosing, frequent hospital visits, and associated after-care costs; it is important to note that only well-trained health professionals can apply intravitreal injections; when administered by untrained or inexperienced subjects, the risk of producing injury and the incidence of application-related adverse effects increases [17,18,19,26,27,28,29,30]. Periocular TA injections, which include suprachoroidal, subtenon (inferotemporal or superotemporal), and subconjunctival injections, are another example of ocular drug delivery. These routes of administration have proven to be safer options than intravitreal injections due to the fact they do not penetrate the eye, and thus, involve fewer anatomical structures when administered [31,32,33]. However, associated effects such as increasing intraocular pressure (IOP), or the still ongoing clinical research have reduced its use in clinical practice [7,8,9,26,27]. Systemic TA administration, including oral or parenteral, has been in disuse due to lower eye drug concentrations and an increase in the incidence of adverse systemic effects [10,26,27].

## 3. Biological Effects of TA

TA’s anti-inflammatory and analgesic properties are attributed to its ability to induce lipocortins, these proteins have been shown to reduce leukocyte chemotaxis, control biosynthesis, and inhibit the release of arachidonic acid from the phospholipid membrane, which is one of the most important common precursors of potent inflammatory cell mediators including prostaglandins and leukotrienes [4,34]. Corticosteroids also regulate gene expression in the posterior segment of the eye, influencing factors such as VEGF, TNF-α, and inflammatory chemokines and inducing the expression of anti-inflammatory factors including the pigment epithelium-derived growth factor. Some studies have shown that TA significantly inhibits the expression of TNF-α, interleukin 1β (IL-1β), thromboxane B2 (TxB2), and leukotriene B4 (LTB4) in a dose-dependent manner. Additionally, TA seems to reduce matrix metalloproteinase expression and downregulate intercellular adhesion molecule 1 (ICAM1) in choroidal endothelial cells [4,35,36]. Corticosteroids are effective in treating edematous and proliferative diseases by targeting abnormal cell proliferation and restoring blood-retinal barrier function. [37]. Valamanesh et al. have studied TA effects on retinal endothelial cells (RPE) in vitro and its potential vascular toxic effect when injected into the vitreous cavity of rats. These authors have demonstrated that TA had induced a time-dependent reduction of retinal endothelial cells’ viability and necrosis, while exposure to lower concentrations for 3 to 5 days had induced caspase-independent apoptosis. It was demonstrated that TA had mostly affected the choroidal vasculature with a reduction of choroidal thickness and had increased the avascular areas of the choriocapillaris. In summary, experiments performed on RPE cells have shown that TA has downregulated the basal expression of COX-2 and VEGF [38].

## 4. Triamcinolone Acetonide Formulations and Adverse Events

### 4.1. Preserved and Preservative-Free Triamcinolone Acetonide Formulations

Studies on animal models have reported retinal toxicity after the intravitreal administration of different preserved corticosteroid formulations, including Celestone Soluspan^®^ (betamethasone sodium phosphate), Depo-Medrol^®^ (methylprednisolone acetate), Decadron^®^ (dexamethasone sodium phosphate), and Decadron L.A^®^ (dexamethasone acetate). The adverse effects include the elevation of intraocular pressure, cataract-genesis, and potential cytotoxicity to ocular structures such as photoreceptors and RPE cells [10,39,40]. Kenalog-40^®^ (Bristol Myers Squibb, New York, NY, USA) is the most frequently applied ocular steroid in humans; it has been used off-label for the treatment of retinal diseases. Each milliliter of the formulation provides 40 mg of TA with sodium chloride for isotonicity, 0.99% benzyl alcohol as a preservative, 0.75% carboxymethylcellulose sodium, and 0.04% polysorbate 80. Sodium hydroxide or hydrochloric acid may be present to adjust pH to 5.0–7.5. Benzyl alcohol and polysorbate 80 have been associated with hypersensitivity and allergic reactions following intramuscular and intra-articular injections. Polysorbate 80 has also been described to trigger hemolytic and anaphylactoid reactions following its injected administration [7,41]. Investigators analyzed the ocular toxicity of TA vehicles on rabbit models, revealing adverse effects such as elevated IOP and increased lens density [42,43]. Intravitreal administration of benzyl alcohol resulted in decreased amplitudes of electroretinogram waves, suggesting severe retinal damage. Structural changes in the retina, including the loss and shortening of external segments and photoreceptors, were also observed. Histological examination indicated severe damage to all retinal layers near the Kenalog^®^ (Bristol Myers Squibb, New York, NY, USA) injection site [44,45]. In humans, Yeung et al. found that intraocular TA in a concentration of 1 mg/mL resulted in cytotoxicity to both human glial cells and human RPE cells [46]. The evidence of retinal toxicity secondary to the compounds in preserved TA formulations such as benzyl alcohol led to an urgent need to find alternative compounds and to create preservative-free formulations. In 2006, Bitter and colleagues developed a simple, reproducible method to produce stable, preservative-free TA (PF-TA) suspensions that can be successfully applied intravitreally [20]. Subsequently, Kim et al. evaluated the safety and pharmacokinetics of the preservative-free formulation of TA in intravitreal injections in rabbits. Electroretinograms were found to remain normal over time, and histopathological analysis showed normal ocular tissues. The half-life of PF-TA in the vitreous was between 24 and 39 days [41]. Current Preservative-free formulations approved for intraocular use include Triesence^®^ (Alcon Laboratories INC, Geneva, Switzerland.), Trivaris^®^ (Allergan Inc, Dublin, Ireland.), Taioftal^®^ (Sooft, Rome, Italy), and ATLC^®^ (Laboratorios Grin, Mexico City, Mexico). Trivaris^®^ (Allergan Inc, Dublin, Ireland.), and Triesence^®^ (Alcon Laboratories INC, Geneva, Switzerland.), have been recently approved by the FDA for ophthalmic use in the treatment of sympathetic ophthalmia, temporal arteritis, uveitis, and ocular inflammatory conditions unresponsive to classical topical corticosteroids. Available commercial TA formulations are listed in Table 1.

### 4.2. Increased Intraocular Pressure as an Adverse Effect following Intravitreal TA Administration

Adverse effects in the setting of intravitreal TA administration such as ocular hypertension, cataract formation, and infectious or sterile endophthalmitis have been previously reported, with ocular hypertension being the most frequently described [18,30,47,48,49,50,51]. High intraocular pressure has been one of the main concerns regarding the adverse effects of intravitreal drug administration. Several clinical trials studying the effects of intravitreal TA have reported an increase in intraocular pressure, regardless of the dose used. In a previous study, 113 eyes were treated with 4 mg of intravitreal TA; an increase in intraocular pressure (IOP) of 5 mmHg in 30% of the eyes and more than 10 mmHg in 11% of them during the first 3 months after the initial injection was found [51]. Another study, which included 71 patients with 25 mg intravitreal TA treatment, reported an increased IOP in 52% of the eyes [48]. Furthermore, Gillies et al. evidenced, during a clinical trial where 75 eyes were treated with 4 mg intravitreal TA, 28% of the eyes required treatment with topical hypo-tensors. The most significant IOP elevation was found at 6 weeks along with signs of cataract progression in the treated group [52]. These findings demonstrate the relevance IOP holds as an adverse effect following intravitreal TA administration.

## 5. Triamcinolone Acetonide Ophthalmic Applications 

In the field of ophthalmology, TA has been studied for at least fifty years. Based on experimental studies, clinical observations, and pathogenic considerations, Machemer et al. suggested the intravitreal delivery of steroids to locally suppress intraocular inflammation, the proliferation of cells, and neovascularization. They demonstrated, for the first time, the safety of intravitreal TA injection supported by a lack of signs of ocular toxicity on electroretinography, electron microscopy, and IOP measures [3,53]. 

TA’s characteristics have turned the drug into a potential therapeutic ally in the ophthalmology field; it has previously been tested for its application in the management of different pathologies such as Neovascularization, Age-related Macular Degeneration, Diabetic Macular Edema, Uveitis, Cystoid Macular Edema, Edema Secondary to Retinal Vein Occlusion, and other ocular pathologies.

### 5.1. Neovascularization

TA effects in neovascularization have been documented in human patients. Proliferative diabetic retinopathy and iris neovascularization were treated by applying 25 mg intravitreal TA before cataract surgery, and the regression of iris neovascularization was found within the first 5 postoperative weeks [54]. The same authors evaluated the safety and efficacy of 25 mg of intravitreal TA as an adjunctive treatment in vitrectomy for proliferative diabetic retinopathy, including a group of 32 eyes and a control that only received *pars plana* vitrectomy. Follow-up occurred within a mean of 5 months, and no significant differences were found in terms of complications or improvement in visual acuity [55]. Furthermore, Maggio et al. reported a case of inflammatory choroidal neovascularization treated with two transtenon retrobulbar injections of TA. The treatment resulted in a regression of inflammatory signs and a reduction of neovascular activity [56]. 

### 5.2. Age-Related Macular Degeneration

TA has been studied for several years as a therapeutic tool to treat age-related macular degeneration showing promising results. An 18-month follow-up study with 30 eyes treated with 4 mg of TA reported decreased exudation and visual improvement in 87% of patients [57,58]. In a larger sample of 151 eyes with choroidal neovascularization associated with AMD, a single 4 mg dose of TA led to smaller neovascular membranes compared to the control group after 3 months, although there was no significant difference in severe visual loss during the first year [59]. In another study, 71 eyes with exudative AMD received intravitreal injections of 25 mg of TA, resulting in a significant improvement in visual acuity after a 7-month follow-up [60,61]. Patients with occult subfoveal neovascularization also experienced increased visual acuity after receiving intravitreal injections of 25 mg of TA [62]. Another trial in patients with exudative age-related macular degeneration demonstrated increased visual acuity in patients treated with TA. The main side effect observed in this study was increased intraocular pressure [63]. 

Sheng et al. conducted a prospective study on 102 eyes with age-related macular degeneration to assess the clinical efficacy and adverse effects of ranibizumab alone or in combination with TA. IOP CMT, BVCA, and interleukin levels were evaluated, IOP remained stable through follow-up, and macular thickness followed a decremental pattern over time and had lower values in the combination group. Visual acuity improved over time in both groups, showing better results in the combination group. Levels of IL-1, IL-6, and IL-8 were reduced in both groups, and there was a marked increase in the concentration of IL-2 in the combination group [64]

### 5.3. Diabetic Macular Edema

TA has been tested as a therapeutic approach to diabetic macular edema with several positive outcomes [65]. Intravitreal TA administration led to improved visual acuity and reduced macular thickness in eyes unresponsive to laser photocoagulation [66]. Significant visual acuity improvement was reported in a group of eyes treated with 25 mg of intravitreal TA [67]. In patients with bilateral macular edema, 4 mg of TA showed effectiveness in reducing macular thickening and improving visual acuity [68,69]. Comparisons between 2 mg and 4 mg TA injections showed no significant differences in central macular thickness, visual acuity, and intraocular pressure [63]. Case reports and studies demonstrated improved visual acuity after intravitreal TA injections in cases of refractory macular edema [70,71,72]. In a larger study, 20–25 mg of intravitreal TA led to a temporary increase in visual acuity in eyes with diffuse macular edema [73]. A study on different doses of intravitreal TA found a more pronounced and lasting response with 13 mg compared to lower doses [74]. A 2-year follow-up study showed improved visual acuity and reduced macular thickness with 4 mg of TA treatment [75]. In cases of laser-resistant diabetic macular edema, intravitreal TA (4 mg) was found to be effective [76]. Other studies reported improvements in retinal thickness, hard exudates, fluorescein leakage, and visual acuity with 4 mg of intravitreal TA [77,78]. 

In a retrospective study, posterior subtenon triamcinolone (PSTA) injection for the treatment of DME in vitrectomized eyes was demonstrated to lower central macular thickness (CMT) and increase best-corrected visual acuity (BCVA) [79]. Additionally, the efficacy and safety of suprachoroidal and intravitreal injections of triamcinolone acetonide in pseudophakic patients with refractory DME caused due to epiretinal membrane (ERM) were compared in a randomized clinical trial. No significant variations in BCVA were found across groups at baseline, one-, or three-month post-injection. In terms of mean central foveal thickness (CFT), no significant changes were seen between the two groups at baseline or the first month, although CFT was considerably reduced in the SCTA group in the third month [31]. Another group of researchers evaluated the efficacy and safety of injecting 4 mg/0.1 mL of triamcinolone into the suprachoroidal area with a custom-made needle to treat diabetic macular edema following pars plana vitrectomy (PPV). The findings revealed an increase in visual acuity and a marked reduction of CFT with no cataract progression in the phakic eye. During the 8-week follow-up period, no increase in intraocular pressure or adverse effects were observed [80].

### 5.4. Uveitis and Cystoid Macular Edema

Treatment with TA for Uveitis and Cystoid Macular Edema has been a subject of study. Suprachoroidal injection of TA is efficient in treating patients with uveitis complicated with macular edema. Patients have shown clinical improvement in vision after administration [81]. Likewise, Hanif Et al. reported that a single dose of suprachoroidal injection of triamcinolone acetonide for the treatment of macular edema secondary to uveitis is safe and efficacious [82]. In pediatric populations, subtenon TA injection with topical anesthesia was well-tolerated and shown to be a reasonable alternative for the short-term treatment of uveitis [83]. 

A clinical trial in which six patients with chronic uveitic cystoid macular edema (CME) were injected with 2 mg of TA into the vitreous cavity demonstrated visual acuity improvement [84]. Moreover, Young et al. presented the safety and efficacy of 4 mg intravitreal TA in the treatment of inflammatory CME resistant to other therapies, within a 12-month follow-up in a six-patient sample [85]. Additionally, another study reported an increase in visual acuity within 5 weeks of treatment with 20 mg intravitreal in a patient with chronic bilateral idiopathic uveitis [86]. A significant improvement in visual acuity and CMT was observed in eyes with subtenon TA for Cystoid Macular Edema due to Retinitis Pigmentosa unresponsive to Carbonic anhydrase inhibitors [87]. Three eyes with pseudophakic CME after uncomplicated cataract surgery were studied after being treated with 8 mg of intravitreal TA showing promising results at a one-month follow-up, with a decrease in macular thickness and improved visual acuity in all patients; however, this improvement appears to be transient, even after a second injection [88]. Intravitreal triamcinolone and dexamethasone implants were both equally effective in increasing visual acuity in patients with Postoperative Cystoid Macular Edema at a 6-month follow-up; however, macular edema seems to respond more rapidly with intravitreal triamcinolone [89]. Tariq et al. reported that intravitreal injection of filtered modified 2 mg TA is safe, effective, and an inexpensive alternative to antivascular endothelial growth factor (anti-VEGF) agents for patients with Postoperative Cystoid Macular Edema, especially for patients concurrently with DM [90]. A study compared subtenon and intravitreal TA for the treatment of PCME and both achieved significant improvement in vision and CMT with no significant difference between interventions at 3- and 6-month follow-ups [91].

### 5.5. Edema Secondary to Retinal Vein Occlusion

Several studies have tested TA as a therapeutic approach for edema secondary to retinal vein occlusion. Researchers administered 4 mg of intravitreal TA in a patient with macular edema secondary to central retinal vein obstruction, and the results showed an improvement in visual acuity and central macular thickness [92]. The same researchers treated a patient with macular edema secondary to bilateral central retinal vein occlusion by applying an intravitreal injection of 25 mg of TA in both eyes. After a 4-month follow-up period, a significant improvement in visual acuity was demonstrated [93]. In another study, 20–25 mg of intravitreal TA was applied in 10 patients with retinal vein branch occlusion, and a significant increase in visual acuity was demonstrated in the study group [94]. Park et al. studied 10 eyes with CME associated with central retinal vein occlusion and treated them with 4 mg of TA injected into the vitreous cavity, showing an improvement in edema and visual acuity [95]. One trial showed a significant increase in visual acuity after 20–25 mg of intravitreal TA in patients with branch retinal occlusion [94]. 

Recently, a study by Ali et al. suggests that suprachoroidal TA could be well tolerated and efficacious as a mono-treatment of macular edema secondary to retinal vein occlusion [96]. Additionally, the joint treatment of triamcinolone acetonide (TA) with antiangiogenics such as Bevacizumab and Aflibercept has been studied in the management of macular edema secondary to retinal vein occlusion (RVO). This combined approach has shown promising results and is considered a safe and cost-effective alternative to antiangiogenic therapy alone [21,97]. 

A nonrandomized, multicenter study conducted by Adelman et al. compared the efficacy of surgical and medical therapies for the treatment of macular edema associated with RVO. In total, 738 cases of RVO were considered, and *pars plana* vitrectomy with the internal limiting membrane (ILM) peeling therapy alone showed better results in recovering the visual function. TA alone or in combination with anti-VEFG reported modest increases in visual acuity, with the latter alone showing the best non-surgical therapy results [98]. Another group of investigators conducted a comparative multicenter interventional study regarding the effects and efficacy of adding intravitreal dexamethasone implant (DEX) or preservative-free TA to monotherapy with bevacizumab for the treatment of refractory CME secondary to RVO. Central macular thickness (CMT) improved in both groups, whereas visual acuity remained similar from baseline. The frequency and need for intravitreal injections diminished in both groups, with greater results in the DEX group [99].

### 5.6. Other Applications 

In addition to the pathologies previously exposed, TA´s capability of treating different ocular diseases has been described and yet remains a promising research field with opportunities to explore. Kaczmarek et al. demonstrated the antiproliferative properties of TA on human cultured RPE cells, suggesting its effectiveness against proliferative vitreoretinopathy (PVR) treatment without producing cytotoxic effects [100]. Another study compared 16 patients undergoing *pars plana* vitrectomy for the treatment of PVR who were given 10–20 mg intravitreal TA; less inflammation and clearer fundus ophthalmoscopy were found in the study group [101]. 

One study reported two cases of refractory CME secondary to birdshot retinochoroidopathy that were successfully treated with intravitreal injections of 4 mg TA [102]. In another study, 20 mg of intravitreal was applied in 14 eyes diagnosed with neovascular glaucoma; the results included a significant decrease in intraocular pressure and *rubeosis iridis* [103]. Additionally, 20 mg of intravitreal TA was applied to two eyes with pre-phthisic ocular hypotonia, finding elevated intraocular pressure and better visual acuity in both eyes in the 3-month follow-up [104]. Likewise, a case report of a patient with sympathetic ophthalmia who was treated with two injections of 25 mg intravitreal TA in 3 months demonstrated an increment in intraocular pressure and improvement in visual acuity on both occasions [105]. A clinical case was also reported in which 25 mg of intravitreal TA was repeatedly administered to a patient with ischemic ophthalmopathy, totaling three applications. After each injection, significant findings included increased visual acuity, regression of iris neovascularization, clearance of vitreous haze, and an increase in intraocular pressure [106]. 

Recently, Nilforushan et al. conducted a case series study to report the effects of 1 subtenon TA injection on non-resolving or progressive serous choroidal detachment (SCD) following glaucoma surgery. Sixteen patients received a sole 1 mL TA (40 mg) injection and were followed for 6 months [107]. Choroidal detachment was resolved within 4 weeks in all cases and no recurrence was reported. Suprachoroidal TA was shown to be an effective adjuvant treatment for VKH serous retinal detachment, without any serious ocular adverse effects or increases in IOP, causing a significant reduction in CFT and rapid improvement in BCVA when combined with oral steroids [108]. Similarly, Sub-Tenon triamcinolone acetonide injection may be an effective and safe treatment in pregnant women with new-onset Vogt–Koyanagi–Harada disease [109].

## 6. Innovative Triamcinolone Acetonide Administration Systems

The use of TA in different ocular diseases is supported by the findings reported in previous literature. However, as previously mentioned, several adverse effects, principally related to the intravitreal route of administration, are still concerning. Further research and development of new carriers that will allow deeper tissue penetration, avoidance of intravitreal injections, and lasting effects of the compound are required to maximize the beneficial effects of triamcinolone administration and reduce the incidence of ocular adverse reactions. The main innovative TA administration approaches in posterior segment ocular disease are intravitreal implants and nanosystem-based drug delivery formulations (nanocarriers). Intravitreal implants consist of either injectable or surgically adhered delivery systems to the vitreous chamber of the eye; they can offer a continuous and sustained release of solutions into intermediate and posterior segments of the eye. Nanocarriers consist of employing materials of 1 to 100 nm as vehicles for ocular drug delivery, improving drug solubility, suffering less metabolic degradation, and decreasing dosing frequency, providing better drug targeting [26]. 

### 6.1. Intravitreal Implants

At the beginning of the 21st century, before preservative-free formulations of TA were developed, anti-angiogenic therapy was proposed as an effective new pharmacological approach for vitreoretinal diseases, reorienting the gold standard and removing TA from treatment regimens. Although steroids were considered a second-line treatment, research efforts were directed toward developing intraocular corticosteroid implants, two of which have already been approved for clinical use: One containing fluocinolone acetonide (Retisert^®^; Bausch and Lomb, Rochester, NY, USA), approved in April 2005, and the other one containing dexamethasone (Ozurdex^®^, Allergan, Dublin, Ireland), approved by the FDA in 2009. Intravitreal implants can be categorized into two different types, which vary in their specific characteristics: Biodegradable (BI) and non-biodegradable (NBI) implants. Non-biodegradable implants consist of a drug reservoir within a permeable membrane made up of a non-degradable polymer, ethylene vinyl acetate (EVA), polyvinyl alcohol (PVA), or polysulfone capillary fiber (PCF). These implants require surgical implantation and removal or replacement once the drug reservoir is empty. Some complications arising from their use include vitreous hemorrhage, retinal detachment, epiretinal membrane formation, or dissolution of the implant. Non-biodegradable intravitreal implantation can also be generally performed in an office setting following aseptic protocols and with adequate equipment at disposal. I-vation^®^ (SurModics, Eden Prairie, MN, USA) is an example of a helically shaped NBI made up of a PVA-EVA reservoir containing 0.925 mcg of triamcinolone acetonide. It is indicated for diabetic macular edema and lasts for 2 years. It is surgically fixed at the *pars plana* through a small incision (<0.5 mm) *pars plana* sclerotomy [110,111]. 

Biodegradable implants (BI) are made up of biocompatible and degradable materials that disintegrate over time; hence, they do not require the necessity of a replacement or surgical removal. Polylactic acid (PLA), polyglycolic acid (PGA), poly (lactic-co-glycolic acid) (PLGA), and polycaprolactones are the primary polymers used. BIs can be formulated as either discs, rods, or microparticles, and carry increased flexibility in comparison to NBIs [110,112]. The Ozurdex^®^ DEX implant (Allergan Inc., Dublin, Ireland) is an FDA-approved dexamethasone-formulated BI for the treatment of retinal vein occlusion-associated macular edema and non-infectious posterior uveitis. It is formulated with dexamethasone within a PLGA matrix slowly degrading into lactic and glycolic acid. It can provide sustained drug release for up to 6 months [111,113]. Currently, no BI containing triamcinolone acetonide has been approved [112].

### 6.2. Nanocarriers

Nanocarriers are classified into three main groups: Polymeric, non-polymeric, and lipid-based nanocarriers, which in turn are subdivided into more specific groups. The polymeric nanocarrier classification includes four main branches: The nanomicelles, the polymeric nanoparticles, dendrimers, and nanogels. On the other hand, two nanomolecules, gold nanoparticles and mesoporous silica, are included in the non-polymeric nanocarrier group. Finally, lipid-based nanocarriers refer to the different subgroups including the emulsion-based, vesicle-based, and particulate systems [26]. One of the benefits of these novel types of approaches relies on the avoidance of systemic administration, reducing the need for high drug concentrations. There exist different nano-system-based TA eye delivery formulations, all of which vary in pharmacokinetics, chemical, morphologic characteristics, and research applications. Among these, dendrimers, polymeric nanoparticles, Nanostructured Lipid Carriers (NLCs), Solid Lipid Nanoparticles (SLNs), and liposomes have shown interesting results. A summary of TA-reported nanocarriers is provided in Table 2.

Dendrimers are nanocarriers that have multiple functional groups on the surface that can be covalently linked to different therapeutic agents and have been widely explored as drug and gene delivery vehicles. The fourth-generation hydroxyl polyamidoamine (PAMAM) dendrimer has shown significant potential for improved, targeted cellular delivery without toxicity and immunogenicity in animal models. Additionally, it has been reported that the conjugation of hydrophobic drugs such as TA to the hydroxyl PAMAM dendrimers increases aqueous solubility, enabling them to be easily formulated in saline for administration. A study by Cho et al. reported that, following intravitreal injection in a mouse model of oxygen-induced retinopathy (OIR), dendrimer-conjugated TA (D-TA) exhibited selective localization and sustained retention in activated microglia in disease-associated areas of the retina, suppressed inflammatory cytokine production, microglial activation, and preretinal neovascularization, and ameliorated OIR-induced neuroretinal and visual dysfunction [120]. Lezzi et al. demonstrated that one intravitreal injection of 1 mg of Fluocinolone acetonide (FA) conjugated with 7 mg of the PAMAM dendrimer was able to arrest retinal degeneration, preserve photoreceptor outer nuclear cell counts, and attenuate activated microglia in a rat retinal degeneration model [122].

SLNs are nanosized lipid carriers based on a physiological, biodegradable, and biocompatible lipid matrix (stabilized by surfactants) composed of 0.1 to 30% solid fat, which is dispersed in an aqueous phase; they were first patented in the 1990s [123,124]. NCLs are considered an improved second generation of SLNs due to their composition containing a controlled nanostructure of the lipid matrix. The process of mixing solid lipids with spatially incompatible liquids gives rise to the chemical formation of special nanostructures with the incorporation of the surrounding molecules, which possess sustained pharmacological release properties [125]. A group of researchers developed a topical SLN-containing gel formulation loaded with TA. The in vivo examination of rabbit eyes has shown an improved precorneal residence time and higher concentrations of the drug in aqueous humor, vitreous humor, and cornea at 6 h, compared to a free suspension of TA or simple SLN [117]. Another study used rabbit scleral tissue to which a TA-NLC formulation was applied and furtherly analyzed. The ability to diffuse through the sclera with a sustained drug-release profile following zero-order kinetics was demonstrated. Importantly, strong tissue binding was observed, providing evidence of the formation of a drug reservoir [126]. Similarly, in an in vivo study with mice, the ability to deliver lipophilic compounds to the posterior segment of the eye through corneal and non-corneal routes was demonstrated by a TA-encapsulated NLC eye drop instillation [121].

Polymeric nanoparticles (NPs) are particles within the size range from 1 to 1000 nm and can be loaded with active compounds entrapped within or surface-adsorbed onto the polymeric core. They have shown enormous potential for the targeted delivery of drugs for the treatment of several diseases [127]. A study by Tatke et al. developed sustained-release triamcinolone acetonide (TA)-loaded polymeric matrix films for ocular delivery by applying melt-cast technology, and polyethylene oxide (PEO) as the polymer matrix. The in vivo studies in albino rabbit eyes demonstrated that significantly higher TA levels were seen in the anterior and posterior segments of the eye at the end of 6 h with the PEO films compared with a 4% *w*/*v* TA suspension (TA-C). Therefore, the PEO-based polymeric films delivered TA into the back of the eye efficiently and for prolonged periods. [128]. Ocular pharmacokinetic studies in rabbits showed significantly higher and sustained vitreous humor exposure of a Triamcinolone acetonide (TA)-loaded dual responsive in situ gelling system using reacted tamarind seed xyloglucan (RXG) (thermoresponsive) and kappa-Carrageenan (κ-CRG) (ion-sensitive) polymers compared to a hydroxypropyl-β-cyclodextrin based aqueous suspension of TAA [129].

Another type of nanocarrier that has gained interest in the ophthalmology field is liposomes, which were first described by Alex Bangham in the 1960s. In 1981, Samolin et al. investigated the role of liposomes in ophthalmic drug delivery [130]. Since then, several liposome formulations have been tested on their ability to release drugs into the deep layers of the eye, and multiple administration routes have surged, such as topical, subconjunctival, intravitreal, and intravenous. Liposomes consist of single or multiple vesicles composed of a phospholipid bilayer with a structural resemblance to the cell membrane that forms small spheroids able to carry both hydrophilic and lipophilic drugs through chemical naturally occurring reactions. Phospholipids and water combine to form a biphasic sphere with a hydrophilic and hydrophobic cavity. Aggregation of the hydrophobic segments during sphere formation traps water-soluble drugs, while those that are fat-soluble are incorporated into the phospholipid layer [131]. Jin Li et al. developed a formulation of Triamcinolone Acetonide-Chitosan-Coated liposomes (TA-CHL), which demonstrated high entrapment efficiency and was described to provide effective drug delivery to the posterior segment of the eye via drop instillation in animals [132]. The same research group demonstrated in preclinical studies conducted on a laser-induced retinal edema rat model that TA-CHL eye drops resulted in long-lasting beneficial therapeutic effects [133]. Another study conducted on rat models, which received topical administration of a formulation with Chitosan-Coated Liposomes (CCL) encapsulating TA, demonstrated penetration through the corneal mucosal barrier and drug accumulation in the vitreous body [134]. Formica et al. conducted a study that aimed to address the limitations associated with the ocular use of triamcinolone acetonide (TA) as an alternative formulation using lipid nanocapsules (LNCs). The group successfully prepared triamcinolone acetonide-loaded lipid nanocapsules (TA-LNCs) relying on a phase-inversion temperature process without the need for external excipients. TA-LNCs demonstrated both in vitro and in vivo efficacy. In vitro experiments demonstrated TA-LNCs at 0.1 µg/mL to be non-toxic to human corneal epithelial cells while effectively reducing interleukin-6 concentrations, indicating high anti-inflammatory activity. Results of an endotoxin-induced uveitis rabbit model showed TA-LNCs significantly attenuated clinical signs of an inflammatory response, where a single 100 μL subconjunctival TA-LNCs dose at concentrations of 250 µg/mL reduced fogginess on aqueous humor examination, highlighting their therapeutic potential [135]. Another research group described the characterization of a TA liposome-containing formulation (TA-LF). The authors concluded the solution met the stability requirements for its intended use in the treatment of inflammatory conditions of the eye. To prove that TA topically administered by TA-LF was able to reach the vitreous humor and intraocular structures, in vivo studies in rabbits were performed. The capacity of diffusion of topical TA-LF through corneal tissue was evaluated with the help of rabbit corneal tissue and diffusion chambers, with results showing that TA-LF could cross corneal tissue and reach the vitreous humor [6]. The same authors described that, following the instillation of one drop of TA-LF (6 times a day for 14 days) in one eye, TA was detected in different ocular tissues as the conjunctiva, cornea, lens, retina, aqueous, and vitreous humor [6]. 

As previously exposed, several types of nanoparticles have shown promising results in preclinical studies; however, only a few have reached clinical trials. A phase I clinical assay evaluated the safety and local tolerability of TA-LF upon repeated-dose topical application to one eye in twenty healthy volunteers. TA-LF was well tolerated during the study period. No systemic adverse effects (AEs) or serious AEs were reported. None of the 20 patients showed significant changes in IOP, BCVA, contrast sensitivity, or CFT, and none of the patients required IOP-lowering drug treatment. Endothelial cell concentration and retinal condensation were unaltered [114]. The efficiency profile of topical TA-LF has been reported in different posterior segment affections. TA-LF showed improved visual acuity and diminished central foveal thickness (CFT) in patients with DME [114], in patients with macular edema secondary to retinal vein branch-occlusion, and in patients with pseudophakic CME [116,136]. Interestingly, non-serious adverse events were reported in these clinical trials. Another study found that the administration of TA-LF after cataract laser-assisted surgery improved contrast sensitivity and prevented pseudophakic CME [115]. More recently, the efficacy and safety of topical TA-LF when used as an adjunct to intravitreal administration of ranibizumab (RBZ) in treatment-naïve patients with neovascular age-related macular degeneration (nAMD) were reported. No significant differences were observed between RBZ/TA-LF and RBZ groups regarding BCVA or CFT. However, the mean number of RBZ injections was significantly lower in the RBZ/TA-LF group, with 2.5 ± 1.4 versus 6.1 ± 1.3 for intravitreal injections [137]. 

A summary of clinical studies using novel TA liposome formulations as primary or adjuvant therapy for retinal diseases is provided in Table 3.

## 7. Summary and Conclusions

Intravitreal corticosteroids have turned into one of the most effective and widely used molecules for the treatment of different vitreoretinal disorders. TA has special characteristics including long-lasting activity, an extensive capacity of reducing the inflammatory response, edema formation, leukocyte migration, capillary dilatation, fibroblastic proliferation, and collagen deposition, which made it the most common synthetic corticosteroid used in eye-related processes. Considering the findings reported by several studies, the injection of intravitreal TA in different doses is a successful treatment in diverse ocular pathologies including AMD, CME, and Diabetic Macular Edema; however, intravitreal TA administration has several disadvantages such as the need for a trained professional to perform the injection and the potential risk of application side effects. 

Widely described retinal toxicity related to preservative-containing TA formulations has paved the way for developing preservative-free TA formulations, which have been shown to produce less toxicity and damage to ocular tissues. 

Adverse events reported in past preclinical and clinical trials related to intravitreal TA administration suggest the necessity to develop new TA release systems, capable of reaching as much penetration as an intravitreal injection without its related inconveniences and side effects, to use TA as an adjunct to current gold-standard therapies such as antiangiogenics or as a first-line treatment. 

To solve this drug-delivery problem, intravitreal implants and topical nano formulations have been in the scope of the study. From all these novel administration approaches, topical liposome TA formulations have been clinically tested and have shown promising results in the treatment of vitreoretinal diseases and are an exciting prospect for becoming an alternative or replacement for TA intravitreal injections. 

## Figures and Tables

**Table 1:** Available commercial TA formulations.

Name	Formulation	Route	Labeler	Notes
Kenalog-40^®^	40 mg/1 mL	Intravitreal	Bristol Myers Squibb	Contains 0.99% benzyl alcohol as a preservative. Available for off-label use.
Triesence^®^	40 mg/1 mL	Intravitreal, Ophthalmic	Alcon Laboratories INC.	Preservative-free formulation.
Trivaris^®^	8 mg/0.1 mL	Intravitreal	Allergan	Preservative-free formulation. Available as a suspension.
ATLC^®^	40 mg	Intravitreal	Laboratorios Grin	Preservative-free formulation. Available as a 40 mg micronized particles powder; must be reconstituted with 1 mL of injectable solution.
Taioftal^®^	80 mg/mL	Intravitreal	Sooft	Preservative-free formulation.

**Table 2:** Nanocarriers in triamcinolone acetonide ocular delivery.

Nanocarrier	Formulation	Dosage	Type of Study	Findings
Liposomes	2 mg/mL TA (0.2%)	6 times a day	Clinical study: Pilot study	Visual acuity and contrast sensitivity improved over time and central foveal thickness in patients with diabetic macular edema [114].
	2 mg/mL TA (0.2%)	4 times a day for 21 days postoperatively	Clinical study: Phase IIb	Patients improved contrast sensitivity after cataract surgery in comparison to the prednisolone and nepafenac groups [115,116].
Solid lipid nanoparticles (SLN) and in situ gel containing SLNs (TA-SLN-IG)	1 mg/mL TA (0.1%)	1 dose of 50 µL of the TA-SLN and 1 dose of 50 µL of the TA-SLN-IG (0.1% w/v)	Preclinical study: in vivo animal study (rabbits)	TA-SLN-IG showed increased concentrations in ocular tissue compared to TA-SLN, but TA-SLN had better performance than TA in the suspension and TA-C groups. Peak tear concentrations of TA-SLN and TA-SLN-IG were achieved after 1 and 2 h, respectively [117].
Cationic nanostructured lipid carriers (cTA-NLC)	40 mg TA (0.1% of solution)	1 mL of the topical cTA-NLC formulation was applied on the epithelial surface and samples were then drained every hour.	Preclinical study: ex-vivo animal study (goat corneas)	Transcorneal studies showed that 39% of the cTA-NLC permeated within the first 4 h. The remaining showed a slower permeation rate of around 51% of the drug total at the end of 8 h compared to a free TA-suspension formulation, which showed a faster but decremental rate of transcorneal permeation. The highest drug concentration (29%) was achieved at 4 h; at this point, drug concentrations followed a decremental pattern, with a drug concentration of 22% at 8 h [118].
PLGA polymer nanoparticles (PLGA NPs)	PLGA-TA nanoparticles 1% TA/PLGA ratio (w/w) 1/10 2/10 4/10	50 µL of topically applied PLGA-TA nanoparticles 1% every 4 h.	Preclinical study: in vivo animal study (rabbits) and endotoxin-induced uveitis rabbit model (EIU).	Aqueous humor flare was evaluated on the uveitis models. Topical formulations showed a similar inflammation reduction pattern on the EIU models in the first 16 h; past that time, the PLGA-TA nanoparticles showed a more significant and sustained flare reduction in comparison to a free micro-suspension and a subconjunctival TA injection. PLGA-TA nanoparticles showed similar effects to subconjunctival TA injection on the reduction of cell, nitric oxide, protein, and PGE2 concentrations on aqueous humor following LPS administration [119].
Dendrimer-conjugated TA (D-TA)	D-TA (0.5 µg)	1 intravitreal injection of D-TA (0.5 µg) or free TA (0.5 µg)	Preclinical study: in vivo animal study (rats)	D-TA suppresses the formation of pathological neovascular tufts in contrast to free TA at the same dose concentration (0.5 µg). D-TA demonstrated damage-dependent distribution, targeting, and exhibiting colocalization with activated microglia in areas of vaso-obliteration and neovascular formation. Microglial intracellular D-TA signals were mostly exhibited when examined in both VO and NV areas. There was an absence of intracellular fluorescent D-TA in other cell types [120].
Nanostructured lipid carriers (NLC)	TA-NLC (4 µg)	A single drop of TA-NLC formulation (4 µg)	Preclinical study: in vivo animal study (CD1 mice)	The short and long-term stability of TA-NLC in CD1 mice was assessed by high-performance stability analysis using the Turbiscan^®^. The results showed a backscattering of less than 1.5% and during a period of 6 months, anticipated the low tendency of these particles for aggregation during shelf life when stored at room temperature [121].

**Table 3:** Main clinical studies of triamcinolone acetonide liposome formulations in the treatment of different retinal diseases.

Disease	Type of Study	Route and Dosage	Clinical Effects	Adverse Effects	Reference
Post-surgery cystoid macular edema	Clinical Study Phase IIb	Topical Prednisolone (1%) and nepafenac (0.1%) (P + N). OR TA (0.2%) liposome formulation (TA-LF).	Contrast sensitivity symptoms improved over time in the TA-LF group, while the P + N group showed no differences from baseline. A significant reduction in intraocular pressure was achieved in both groups.	No adverse effects were reported.	[115]
	Clinical Study Phase IIb	Topical TA conventional therapy group (0.1% TA) for 21 days postoperative OR TA (0.2%) liposome formulation (TA-LF).	The incidence of macular edema at 6 weeks was higher in the TA group (22%) compared to the TA-LF group (0%).	No adverse effects were reported.	[138]
Neovascular age-related macular degeneration	Clinical Study Phase IIb	Intravitreal and topical Single dose of IVT ranibizumab (0.5 mg) and topical liposome TA formulation (2 mg/mL). OR Intravitreal Three IVT monthly doses of ranibizumab (0.5 mg).	Significant improvement in best-corrected visual acuity (BCVA) and reduction of central foveal thickness (CFT) in both groups, but the combined group needed fewer injections. No significant changes in intraocular pressure in any of the groups.	Mild dryness (25%) and burning sensation (37.5%) were reported in the liposome group.	[137]

## Data Availability

The data presented in this study are available upon request to the corresponding author.
